# Ultra-high dimensional variable selection with application to normative aging study: DNA methylation and metabolic syndrome

**DOI:** 10.1186/s12859-017-1568-1

**Published:** 2017-03-07

**Authors:** Grace Yoon, Yinan Zheng, Zhou Zhang, Haixiang Zhang, Tao Gao, Brian Joyce, Wei Zhang, Weihua Guan, Andrea A. Baccarelli, Wenxin Jiang, Joel Schwartz, Pantel S. Vokonas, Lifang Hou, Lei Liu

**Affiliations:** 1 Department of Statistics, Northwestern University, 2006 Sheridan Road, Evanston, 60201 USA; 2 Department of Preventive Medicine, Northwestern University, 680 N Lake Shore Drive, Chicago, 60611 USA; 3 Center for Applied Mathematics, Tianjin University, 92 Weijin Road, Tianjin, 300072 China; 4 Department of Biostatistics, University of Minnesota, 420 Delaware, Minneapolis, 55455 USA; 5 Department of Environmental Health, Harvard University, 677 Huntington Avenue, Boston, 02115 USA; 6 Department of Preventive Medicine and Epidemiology, Boston University, 801 Massachusetts Avenue, Boston, 02118 USA

**Keywords:** Ultra-high dimensional variable selection, ISIS, elastic net, Bootstrap, Metabolic syndrome, methylation

## Abstract

**Background:**

Metabolic syndrome has become a major public health challenge worldwide. The association between metabolic syndrome and DNA methylation is of great research interest.

**Results:**

We constructed a binomial model to investigate the association between a metabolic syndrome index and DNA methylation in the Normative Aging Study. We applied the Iterative Sure Independence Screening (ISIS) method with elastic net penalty to DNA methylation levels at 484,548 CpG markers from 659 human subjects, and demonstrated that the screening step in ISIS can significantly improve the performance of the elastic net.

**Conclusion:**

The proposed method identifies four CpGs which can be mapped to two biologically relevant and functional genes. Identification of significant CpG markers may potentially have practical implications for disease prevention and treatment.

## Background

DNA methylation is an epigenetic mechanism for regulating gene expression. Chemically, it involves the modification of a cytosine (C) base by adding a methyl group. In adult cells, DNA methylation typically occurs at CpG sites, i.e., regions of DNA where cytosine (C) and guanine (G) bases are linked by a phosphate. It can suppress the expression of neighboring genes without changing the underlying genetic sequence. Methylation has been the most commonly studied epigenetic marker because of its transmissibility during cell division as well as stability in stored and processed blood samples. Deciphering the DNA methylation code will help us predict and prevent diseases [1, 2].

One of the major public health challenges worldwide is the steadily increasing prevalence of metabolic syndrome that follows in the wake of society-wide changes such as urbanization, surplus energy intake, increasing obesity and sedentary lifestyle. The International Diabetes Federation estimates that one-quarter of the world’s adult population has metabolic syndrome [3]. Metabolic syndrome is significantly associated with risks of developing cardiovascular disease and diabetes [4]. Our goal is to explore the associations between metabolic syndrome and ultra-high dimensional DNA methylation markers.

Our motivating example is the Normative Aging Study (NAS), where methylation levels from 484,548 CpG sites were measured in 659 subjects. This paper describes our application of an Iterative Sure Independence Screening (ISIS) method [5, 6] with elastic net penalty [7] to address the ultra-high dimensionality and correlation structure of these methylation markers.

The structure of the paper is as follows. In “Results” section, we use simulations to evaluate the performance of our method and apply it to the NAS data. Then, we give the clinical interpretation of our findings in “Discussion” section. In “Discussion” section, we demonstrate the results of using our method on the NAS data. Finally, in “Conclusions” section, we conclude with a summary discussion and possible directions for future research.

## Methods

### Data

The Normative Aging Study (NAS) is a longitudinal cohort study established in 1963 by the Department of Veterans Affairs [8]. With an initial cohort of 2280 healthy men, NAS is an ongoing project to study the effects of aging on various health issues. Eligibility criteria at enrollment included veteran status; residence in the Boston area; ages 21-80; and no history of hypertension, heart disease, cancer, diabetes, or other chronic health conditions. From 1963 to 1999, 981 participants died and 470 were lost to follow up. Participants were recalled for clinical examinations every 3-5 years. Between March 1999 and December 2013, 802 (96.7%) of the remaining 829 active participants agreed to donate blood, 686 of whom were randomly selected and profiled using the Illumina 450K BeadChip array at up to three follow-up visits separated by a median time interval of 3.5 years (IQR 3.1-5.7). We excluded participants who 1) were non-white or had missing information on race to minimize potential confounding effects of genetic ancestry, or 2) had leukemia diagnosed prior to or during the year of their blood draw as their blood methylation profiles could have been affected. A total of 664 individuals and samples collected at their first blood draw remained for analysis.

DNA samples were extracted from buffy coat using the QIAamp DNA Blood Kit (QIAGEN, Valencia, CA, USA). A total of 500 ng of DNA was used to perform bisulfite conversion using the EZ-96 DNA Methylation Kit (Zymo Research, Orange, CA, USA). To limit chip and plate effects, a two-stage age-stratified algorithm was used to randomize samples and ensure similar age distributions across chips and plates. We randomized 12 samples (sampled across all age quartiles) to each chip, then randomized chips to plates (eight chips per plate). Quality control analysis was performed to remove samples and probes where >1*%* had a detection *p*-value > 0.05. The remaining samples were preprocessed using the Illumina-type background correction [9] and normalized with the dye-bias [10] and BMIQ [11] adjustments.

Beta values for DNA methylation level were calculated as the ratio of the methylated probe intensity to the overall intensity, which can be interpreted as the approximate percentage of methylation. Beta values had a range of 0 to 1, but were severely compressed at the extremes. Consequently, Beta values were converted to M-values through logit transformation, providing insight into the distribution of methylation across the genome difficult to visualize with the raw value [12]. M-values were then used in our analysis. The K-nearest neighbors algorithm was applied in the space of CpG sites to impute missing methylation values [13]. Batch and potential confounding effects of white blood cell subtypes as estimated by Houseman’s method [14] were corrected for using ComBat [15].

Metabolic syndrome is defined as whether at least three of the following five conditions are satisfied (*y*=1) or not (*y*=0):

- Abdominal obesity (waist circumference > 102cm for men);
- High fasting blood sugar (≥ 100mg/dl) or currently taking diabetes medication;
- Reduced HDL cholesterol (< 40mg/dl for men) or currently taking cholesterol medication;
- Hypertension (systolic blood pressure > 130mmHg or diastolic blood pressure > 85mmHg) or currently taking antihypertensive medication;
- Hypertriglyceridemia (≥ 150mg/dl) or currently taking medication for hypertriglyceridemia.

To increase power, in this paper we created a metabolic syndrome index as the number of above satisfied conditions. Five subjects with missing data for the above metabolic syndrome conditions are excluded. The final working dataset includes methylation levels of 659 subjects measured at 484,548 CpG sites.

### Analytical method

Two issues complicate the analysis of DNA methylation data. First, the DNA methylation markers are ultra-high dimensional, i.e., *p*≫*n*. Second, DNA methylation levels measured from probes in close proximity are correlated [16]. For example, in the NAS data, the co-methylation correlation could be as high as 0.98 as the samples were free of cell culture-induced epigenetic changes. It is thus imperative to account for ultra-high dimensionality and high correlation simultaneously. In this paper, we adopt the ISIS approach, an iterative two-step procedure combining the screening and variable selection steps.

Fan and Lv [5] proposed the sure independence screening (SIS) and Iterative SIS (ISIS) methods. Later, Fan et al. [6] extended ISIS to the general pseudo-likelihood framework. In SIS, all predictor variables are first ranked based on their Pearson correlations with the response variable. Then, model selection is conducted using a predefined number of the most highly correlated variables. The goal for ISIS is to rescue some variables among missed variables iteratively by ranking marginal correlations with residuals. It can detect important predictors which are marginally uncorrelated by themselves but jointly correlated with the response. Least absolute shrinkage and selection operator (LASSO), smoothly clipped absolute deviation (SCAD), Dantzig selector, and other methods are used for model selection in [5, 6]. For the analysis in this paper, the elastic net penalty is considered to account for correlated methylation markers.

As a compromise between the ridge and LASSO methods, elastic net enjoys a similar sparsity as LASSO but shrinks together the coefficients of correlated predictors like ridge. It also offers considerable computational advantages over the *L*_*q*_ penalties where *q*∈(0,1) [7, 17, 18]. The elastic net penalty has been used widely to conduct model selection in epigenetic studies. For example, [19] built a predictive model of aging using elastic net combined with a bootstrap approach. [20] also used the elastic net regression model to predict epigenetic age across a broad spectrum of human tissues and cell types.

The screening step in ISIS could reduce the ultra-high dimensional covariates to a manageable number by identifying markers which are marginally correlated with the outcome. As a result, in the variable selection step we can tackle the correlation issue in a much smaller covariate space, in which elastic net is expected to perform well. The iterative procedure can recover variables missed at the screening step. Hereafter we choose a weight coefficient of *w*=0.5, i.e., half LASSO and half ridge penalties.

We will use a binomial model for the ordinal metabolic syndrome index {0,1,…,5} as a response variable (*y*) and methylation levels as predictor variables (**x**). *n* is a sample size and *π*_*i*_ is a probability of having any of the above metabolic syndrome conditions for the *i*th subject.

1 $$\begin{array}{*{20}l} y_{i} & \sim \text{Binomial}(5,\pi_{i}) \quad \text{for }\,\, i=1,\cdots, n. \end{array} $$

2 $$\begin{array}{*{20}l} {}\text{logit}\left(\pi_{i}\right) & = \log\left(\frac{\pi_{i}}{1-\pi_{i}}\right)=\boldsymbol{\beta}^{T}\boldsymbol{x}_{i}, \end{array} $$

3 $$\begin{array}{*{20}l} \hat{\boldsymbol\beta} & = \underset{\boldsymbol{\beta}}{\arg\max} \prod_{i=1}^{n} \left(\begin{array}{c} 5\\ y_{i}\\ \end{array} \right) \pi_{i}^{y_{i}} \left(1-\pi_{i} \right)^{5-y_{i}} \end{array} $$

4 $$\begin{array}{*{20}l} & =\! \underset{\boldsymbol{\beta}}{\arg\max} \prod_{i=1}^{n} \! \left(\!\begin{array}{c} 5\\ y_{i}\\ \end{array}\!\right)\! \left(\! \frac{e^{\boldsymbol{\beta}^{T}\boldsymbol{x}_{i}}}{1+e^{\boldsymbol{\beta}^{T}\boldsymbol{x}_{i}}} \!\right)^{y_{i}}\!\! \left(\! \frac{1}{1+e^{\boldsymbol{\beta}^{T}\boldsymbol{x}_{i}}} \!\right)^{\! 5-y_{i}}\!. \end{array} $$

All methods were implemented in the R programming language. See https://github.com/GraceYoon/ISIS_EN for the R source code and an simple example.

## Results

### Simulation

We will illustrate our method by simulation. R is incapable of generating an ultra-high dimensional correlation matrix (484,548 by 484,548). Therefore, in a similar fashion to [21], the real NAS methylation data set is used as an *n*×*p* design matrix (**X**=(**x**_1_,**x**_2_,…,**x**_*n*_)^*T*^=(*X*_1_,*X*_2_,…,*X*_*p*_)) to take the correlation structure among covariates into account. We randomly generate *y* from a binomial distribution with parameters *m*=5 and *π*(**x**). Then, each element of *y*=(*y*_1_,…,*y*_*n*_) can take an integer value ∈{0,1,2,3,4,5} for the metabolic syndrome index. This yields simulation data the same size as the NAS dataset: *n*=659 and *p*=484,548. We used the following coefficients as true parameters ***β***=(*β*_1_,*β*_2_,…,*β*_*p*_)^*T*^ in the simulation setting which are the estimated coefficients in the actual data analysis:

$$\begin{array}{@{}rcl@{}} \left(\beta_{474287}, \beta_{126564}, \beta_{38487}, \beta_{325547} \right) & = & \left(6.6, 2.2, 3.5, -6.3\right), \\ \beta_{j} & = & 0\ \text{for all other}\ j' \mathrm{s}. \end{array} $$

For ISIS, we need to choose a proper submodel size (*d*) in the screening step, which should be large enough to include the true significant coefficients with a probability approaching 1. According to [5], $d= \Bigl \lfloor \frac {n}{4\log (n)} \Bigr \rfloor $ is recommended for a binary outcome, $d=\Bigl \lfloor \frac {n}{2\log (n)} \Bigr \rfloor $ for count, and $d=\Bigl \lfloor \frac {n}{\log (n)} \Bigr \rfloor $ for a continuous outcome, where *n* is a sample size. Since *y* takes integer values from 0 to 5, we choose two values of *d* here: $d= \Bigl \lfloor \frac {n}{2\log (n)} \Bigr \rfloor = 50$, and $d= \Bigl \lfloor \frac {n}{4\log (n)} \Bigr \rfloor = 25$.

The study by Hannum [19] implemented the elastic net penalty on bootstrap samples, and selected CpG markers which were presented for more than half of all bootstraps. Before that, [22] and [23] proposed Bolasso (bootstrap-enhanced lasso): use LASSO for bootstrapped replications of a given sample, and intersect the supports of the LASSO bootstrap estimates. A softer version of Bolasso selects those variables which are present in a high proportion of bootstrap replications. These papers showed that Bolasso leads to consistent model selection. Along these lines, we generated 100 bootstrap samples of the same size (*n*=659), and used ISIS with elastic net penalty to select the significant methylation markers in each bootstrap sample. Here we show the results from ISIS with elastic net, using two different choices of *d* on 100 bootstrap samples. For comparison, we also list the results estimated by elastic net only (without the screening step) in Table 1.

**Table 1:** Simulation results

Elastic net only	ISIS with elastic net				
		*d*=50		*d*=25	
*j*	freq	*j*	freq	*j*	freq
474287	100	474287	100	474287	100
325547	96	126564	84	126564	87
126564	84	38487	68	38487	66
38487	76	325547	65	325547	54
70756	72	359976	26	359976	18
320060	64	384921	21	384921	18
270466	56	258231	19	425056	14
88446	55	425056	18	258231	12
278727	55	86441	15	292919	11
56822	53	329494	15	324153	11
35978	49	320139	14	329494	11
30499	48	90855	13	264984	9
322963	45	233845	13	320139	9
350509	45	324153	13	430210	8
61038	42	16510	12	358567	7
213264	42	361987	12	16510	6
223673	42	46090	11	44790	6
381178	42	264984	11	46090	6
452998	41	349498	11	233845	6
36696	40	86525	9	86525	5

In all cases, the four nonzero coefficient variables are all correctly selected the most often. However, the elastic net only method (without screening) identified 6 additional false markers (70756, 320060, 270466, 88446, 278727, 56822) in at least half of all bootstrap samples, indicating a poor performance against false positives. In contrast, ISIS with the elastic net has a much wider gap in selected frequencies between true and redundant variables, and none of the redundant markers are selected in more than 1/3 of the 100 bootstrap samples. The results from the two different sizes of *d* are consistent with one another.

We repeated this process 5 times (5 datasets with 100 bootstrap samples for each dataset) with consistent results (available upon request). Moreover, we have conducted simulations with varying weights *w*=0.25,0.75 and 1 (LASSO), under the same simulation setting (available upon request). The results show that a larger *w* results in a sparser model when there is no screening step. However, there is virtually no change for different *w* values in ISIS, demonstrating the robustness of ISIS with respect to the weight chosen.

### Application to NAS data

Similar to the Simulation Section, we generated 100 bootstrap samples from the original data. Table 2 shows the selected markers and frequencies of their selection in the model out of 100 bootstrap samples. Among 484,548 CpGs, our method identifies four CpG sites as being strongly associated with metabolic syndrome.

**Table 2:** NAS data results

Elastic net only	ISIS with elastic net				
		*d*=50		*d*=25	
*j*	freq	*j*	freq	*j*	freq
474287	98	474287	91	474287	91
325547	96	126564	67	126564	57
126564	91	38487	46	38487	54
219492	84	325547	46	325547	53
38487	80	12205	20	12205	30
36730	71	141722	17	467369	24
131967	66	467369	16	141722	22
12205	65	351200	15	213684	17
248438	64	147068	13	402549	16
95930	62	402549	12	433494	16
402549	60	193471	11	55087	15
207644	59	213684	11	147068	15
400141	59	268623	11	33489	14
256046	54	55087	10	343324	14
79189	53	104428	10	206869	13
467369	53	433494	10	95930	12
408183	52	95930	9	193471	12
416044	52	219492	9	219492	12
317479	51	248438	9	248438	12
478992	49	206869	8	317479	12

We also compare our results to those from the elastic net only method [19]. As shown in the left column of Table 2, this method identifies 19 CpGs that appear in more than half of the bootstrap samples, many more than the 4 identified by ISIS. For example, the 4th most-selected CpG by the elastic net only method, *X*_219492_, is listed with very low frequency in both columns representing our method. The iterative screening step in ISIS can therefore improve the performance of elastic net by reducing the chance of false positives in ultra-high dimensional data.

To compare the performances of the resulting models, we used 5-fold cross validation to calculate AUC (Area Under the Curve) of ROC curves. Four folds were taken as training data, which we used to build our model. The remaining fold was used as a test datum to calculate AUC. Since we used metabolic syndrome index as a count variable *y*, we measured multiclass AUC proposed by [24] and the average value over 5 folds is reported. We also present the mean AUC value for binary outcomes for the standard definition of metabolic syndrome, i.e., whether at least three of the five conditions are satisfied (*y*=1) or not (*y*=0). These results are shown in Table 3. We note that even though our model has selected many fewer variables (due to the reduced sample size in the training data), its AUC is higher than the elastic net only method which is subject to false positives.

**Table 3:** 5-fold cross validation for AUC

	Elastic net	ISIS, d=50	ISIS, d=25
The number of selected variables	9.6	1.6	1.6
	(2.51)	(0.55)	(0.55)
Average of AUC	0.6011	0.6219	0.6197
Average of multiclass AUC	0.6249	0.6358	0.6441

## Discussion

Associated gene information for the four CpG markers selected by ISIS with the elastic net method is shown in Table 4. The first three CpGs (cg27243685, cg06500161 and cg01881899) are located in close proximity to one another in the same gene: ABCG1. Two, cg06500161 and cg01881899, are at the South Shore and North Shelf of the same CpG Island, respectively. Pfeiffer et al. [25] identified that higher methylation at cg06500161 was associated with lower high-density lipoprotein (HDL) cholesterol and higher triglycerides. The coefficient estimates ($\hat {\beta }$) in Table 4 are consistent with this association. Moreover, methylation levels in cg06500161 and cg27243685 were found to be negatively associated with ABCG1 transcripts. Hidalgo et al. [26] showed associations between the methylation status of cg06500161 and fasting insulin as well as with HOMA-IR (homeostasis model assessment of insulin resistance), a surrogate marker of insulin resistance. Ding et al. [27] reported that it is the most strongly correlated CpG site with BMI among expression-associated methylation sites within one megabase of any cholesterol metabolism network. Our results are also consistent with functional studies of ABCG1 expression. Kennedy et al. [28] and Frisdal et al. [29] identified that higher expression of ABCG1 is associated with increased fat mass, and that deficiency of ABCG1 reduces triglyceride storage. Together, these findings suggest that ABCG1 expression plays a key role in metabolic syndrome, and that DNA methylation may be substantially involved in this pathway.

**Table 4:** Genes associated with the most frequently selected CpG markers

*j*	NAME	CHR	REFGENE	REFGENE GROUP	$\hat {\beta }_{j}$
474287	cg27243685	21	ABCG1	Body; 5’UTR	6.64901
126564	cg06500161	21	ABCG1	Body	2.20140
38487	cg01881899	21	ABCG1	Body	3.49123
325547	cg17901584	1	DHCR24	TSS1500	−6.31737

cg17901584 is located in the TSS1500 region (from -200 to -1500 nucleotides upstream of transcription start site) in the promoter of the gene DHCR24. Drzewinska et al. [30] showed that methylation of the DHCR24 promoter region affects transcriptional efficiency. DNA methylation mediates transcriptional repression via binding of the methylated DNA-binding protein or preserves the binding of transcription factors to their motifs. In the Bloch (Cholesterol Biosynthesis) pathway, desmosterol is converted into cholesterol by DHCR24 in the final step. Zerenturk et al. [31] and Luu et al. [32] found that modulating DHCR24 activity alters levels of desmosterol which further reduces cellular cholesterol status. Thus, DNA methylation may also affect metabolic syndrome via pathways related to DHCR24.

## Conclusions

Using the ISIS method with the elastic net penalty, our study found four important CpGs associated with the metabolic syndrome index from ultra-high dimensional DNA methylation markers. They are located in two biologically relevant and functional genes. Adding the screening step iteratively to the variable selection method is shown to improve its performance against false positives. In conclusion, the two criteria we used: 50% and a gap in the frequencies in the bootstrap samples yield satisfactory selection results against false positives.

In a practical application, one may set $d = \Bigl \lfloor \frac {cn}{\log (n)} \Bigr \rfloor $ in the screening step and select the value of *c* from a grid using the cross-validated prediction error. The adaptive choice of tuning parameter *c* may lead to improved performance when the sample size is not too large.

In NAS, methylation levels were measured up to three times with a median interval of 3.5 years. Our method could be extended to longitudinal data analysis along the way of [33]. Moreover, we are interested in mediation analysis to determine whether methylation mediates the path from intervention (e.g. diet, physical exercise) to health outcomes, thereby helping understand the underlying biological mechanisms of interventions [34].

This analysis is limited to white male subjects in the NAS study. In the future we will validate our results using other cohorts, e.g., the Coronary Artery Risk Development in Young Adults Study (CARDIA), and further examine the relation between DNA methylation and metabolic syndrome in young and middle-aged, mixed-gender, and multi-racial populations.

## Abbreviations

BMIQ: Beta MIxture Quantile dilation
CARDIA: Coronary artery risk development in young adults study
HDL: High-density lipoprotein
HOMA-IR: HOmeostasis model assessment of insulin resistance
IQR: InterQuartile range
ISIS: Iterative sure independence screening
LASSO: Least absolute shrinkage and selection operator
NAS: Normative aging study
